# Supplementary material for: Changes in the Mechanical Properties of Fast and Slow Skeletal Muscle after 7 and 21 Days of Restricted Activity in Rats
Source: Int J Mol Sci. 2023 Feb 18;24(4):4141. doi: 10.3390/ijms24044141 (PMC9966780; doi:10.3390/ijms24044141)
Supplement: Supplementary file 1 [file ijms-24-04141-s001.zip › ijms-2221290-supplementary.pdf]

Supplementary Materials

Table S1. soleus and EDL active mechanical properties

**soleus**	**C7**	**R7**	**C21**	**R21**
rat mass, g	228,3±16,1	237,8±15,2	291,5±9,3	278,1±5,9
soleus mass, mg	110,4±3,1	103,4±3,1	133,7±3,9	120,5±3,5*<br>(-11%)
soleus normalised mass	0,50±0,02	0,44±0,02<br>(-13%)*	0,46±0,02	0,43±0,01
muscle length, mm	19,6±0,3	18,9±0,3	17,9±0,3	18,0±0,2
CSA mm^2	5,4±0,2	5,1±0,2	7,9±0,3	6,5±0,2
twitch tension, mN	77,0±7,1	70,1±5,3	67,2±6,7	59,0±6,1
twitch/CSA, mN/mm^2	13,8±1,0	13,7±1,2	7,7±1,5	7,9±1,4
TTP, ms	43,4±2,4	50,9±3,3	42,7±2,073	46,5±4,2
HRT, ms	56,4±3,6	68,4±6,1	37,6±2,1	40,4±2,6
tetanic tension, mN	543,1±47,5	520,3±40,6	649,2±36,9	426,8±52,2*<br>(-34%)

tetanic tension/CSA, mN/mm^2	98,2±10,0	98,8±5,8	85,9±5,6	60,2±7,9* (-30%)
**extensor digitorum longus**	**C7**	**R7**	**C21**	**R21**
EDL mass, g	117,1±3,1	112,9±3,1	136,1±3,8	128,1±2,8* (-6%)
EDL normalised mass	0,51±0,01	0,48±0,01 (8%)*	0,47±0,02	0,46±0,01
muscle length, mm	25,3±0,5	26,0±0,5	28,5±0,4	27,1±0,4
CSA mm^2	4,3±0,1	4,1±0,127	4,7±0,1	4,5±0,1
twitch tension, mN	102,9±8,8	113,2±9,4	110,9±9,9	74,1±7,8* (-33%)
twitch/CSA, mN/mm^2	24,0±2,3	28,5±3,0	21,9±1,5	15,2±1,0* (-31%)
TTP, ms	19,7±0,5	24,3±0,8* (-19,1)	17,4±0,9	18,7±1,6

HRT, ms	10,9±0,9	12,7±0,6	7,7±0,4	8,0±1,0
tetanic tension, mN	521,5±31,2	555,8±60,2	941,1±79,9	423,3±62,6* (-55%)
tetanic tension/CSA, mN/ mm^2	121,1±9,2	122,4±23,4	203,2±17,9	93,1±12,5* (-54%)

*CSA – physiological cross-section area, TTP – time to peak tension, HRT – half-relaxation time. C – control, R – restricted activity. * - significant differences from «C» (p<0,05). Data shown as mean±SE In parenthesis data shown as % of control group.*

Table S2 soleus and EDL passive mechanical properties

**soleus**	**C7**	**R7**	**C21**	**R21**
F_p, mN	111,3±23,5	94,3±10,6	62,3±4,5	80,0±5,8* (+29%)
F_s, mN	57,4±8,2	51,6±4,2	49,2±5,0	52,9±3,7
F_p/CSA, N/cm^2	2,0±0,3	1,8±0,2	0,8±0,3	1,2±0,09* (+44%)
F_s/CSA, N/cm^2	1,0±0,1	1,0±0,09	0,6±0,04	0,9±0,04* (+50%)
E1, N/cm^2	3,8±0,9	3,3±0,5	1,1±0,1	1,9±0,2*

				(+82%)
E2, N/cm^2	4,1±0,5	4,0±0,3	2,3±0,2	3,5±0,1* (+50%)
**extensor digitorum longus**	**C7**	**R7**	**C21**	**R21**
F_p, mN	141,7±10,6	124,0±11,0	284,4±29,7	167,9±16,7* (-41%)
F_s, mN	70,4±8,7	54,2±4,5	119,9±8,6	81,6±6,5* (-32%)
F_p/CSA, N/cm^2	2,8±0,1	2,4±0,2	6,3±0,8	3,8±0,5* (-40%)
F_s/CSA, N/cm^2	1,3±0,1	1,1±0,1	2,6±0,3	1,8±0,2* (-33%)
E1, N/cm^2	6,2±0,3	5,4±0,5	15,9±1,9	8,2±1,3* (-48%)
E2, N/cm^2	5,2±0,4	4,2±0,3	10,5±1,1	7,0±0,7* (-33%)

*Fp – peak tension, Fs – steady tension, F/CSA –stiffness, E1 and E2 – Young's modulus 1 and 2. C – control, R – restricted activity. * - significant differences from «C» (p<0,05). Data shown as mean±SE In parenthesis data shown as % of control group.*

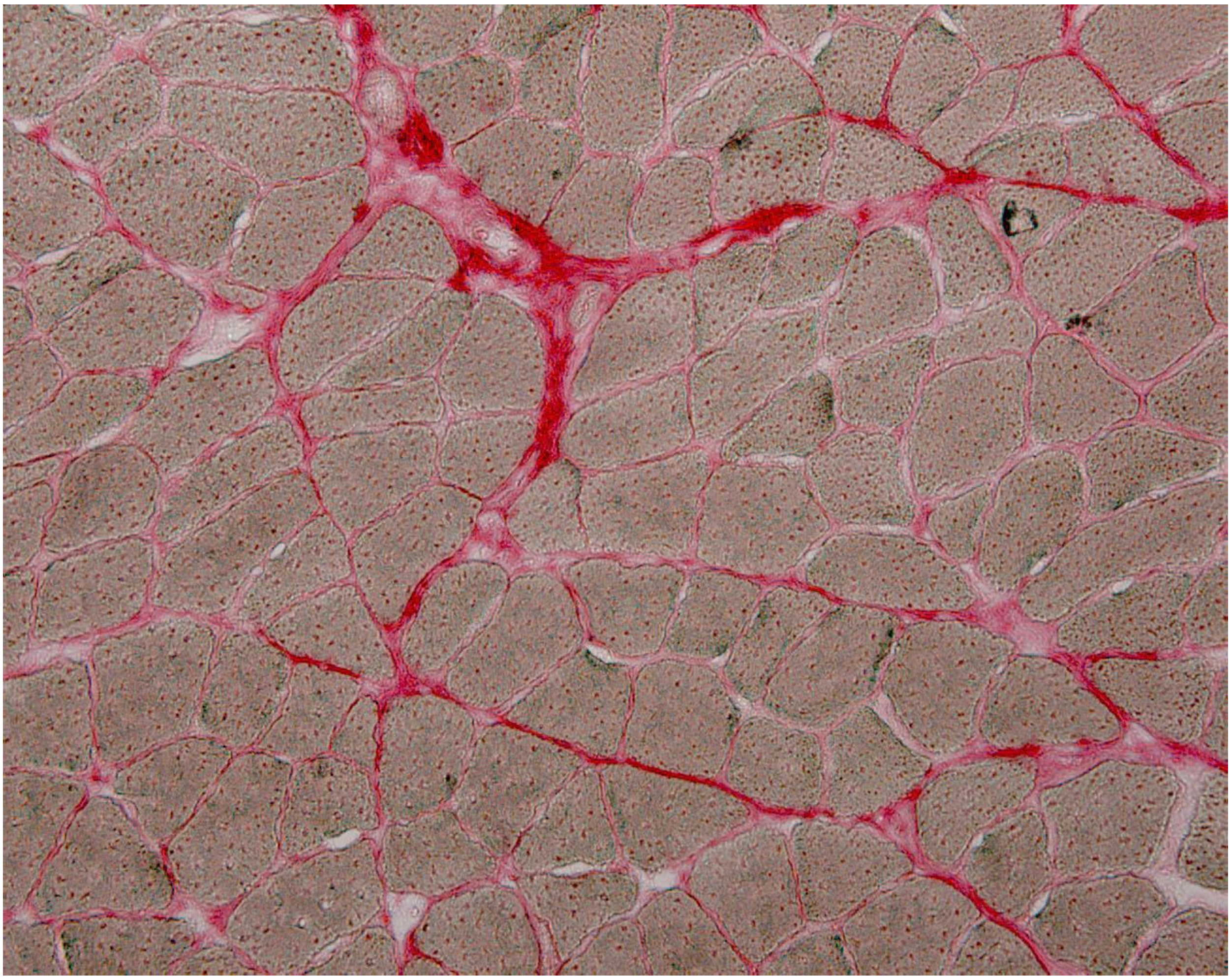

Figure S1. Representative image of collagen staining. Soleus Control

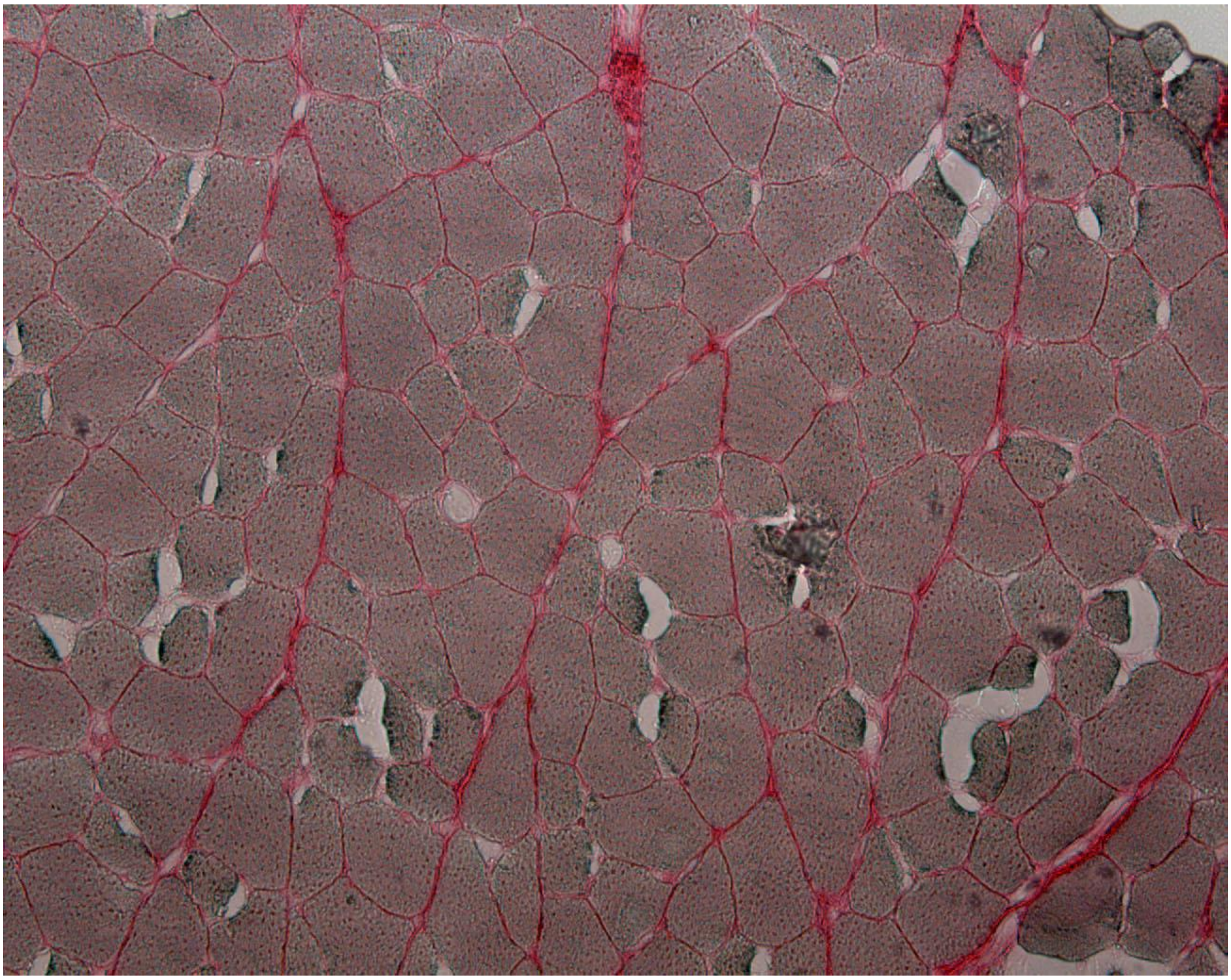

Figure S2. Representative image of collagen staining. EDL Control

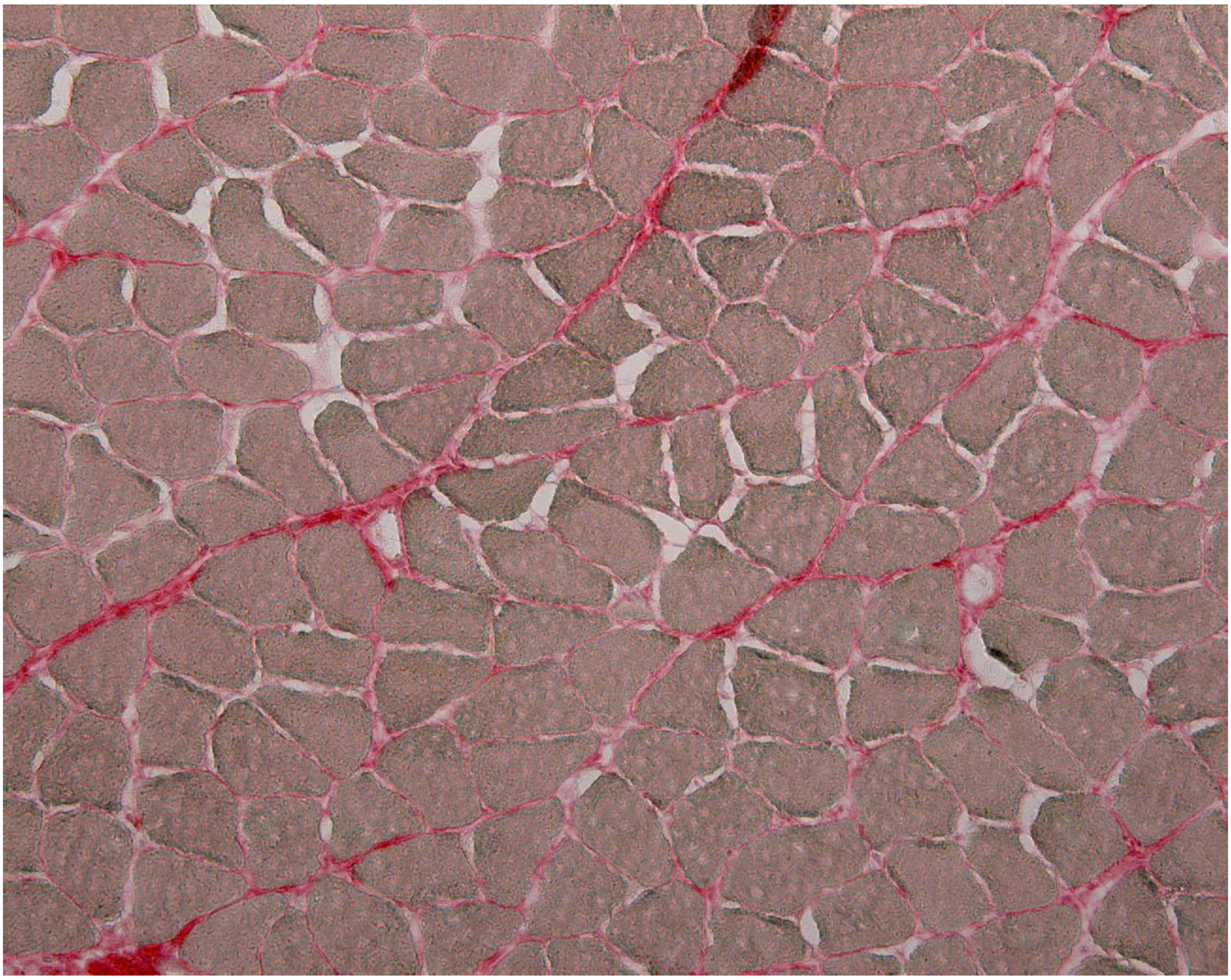

Figure S3. Representative image of collagen staining. Soleus 7-day movement restriction

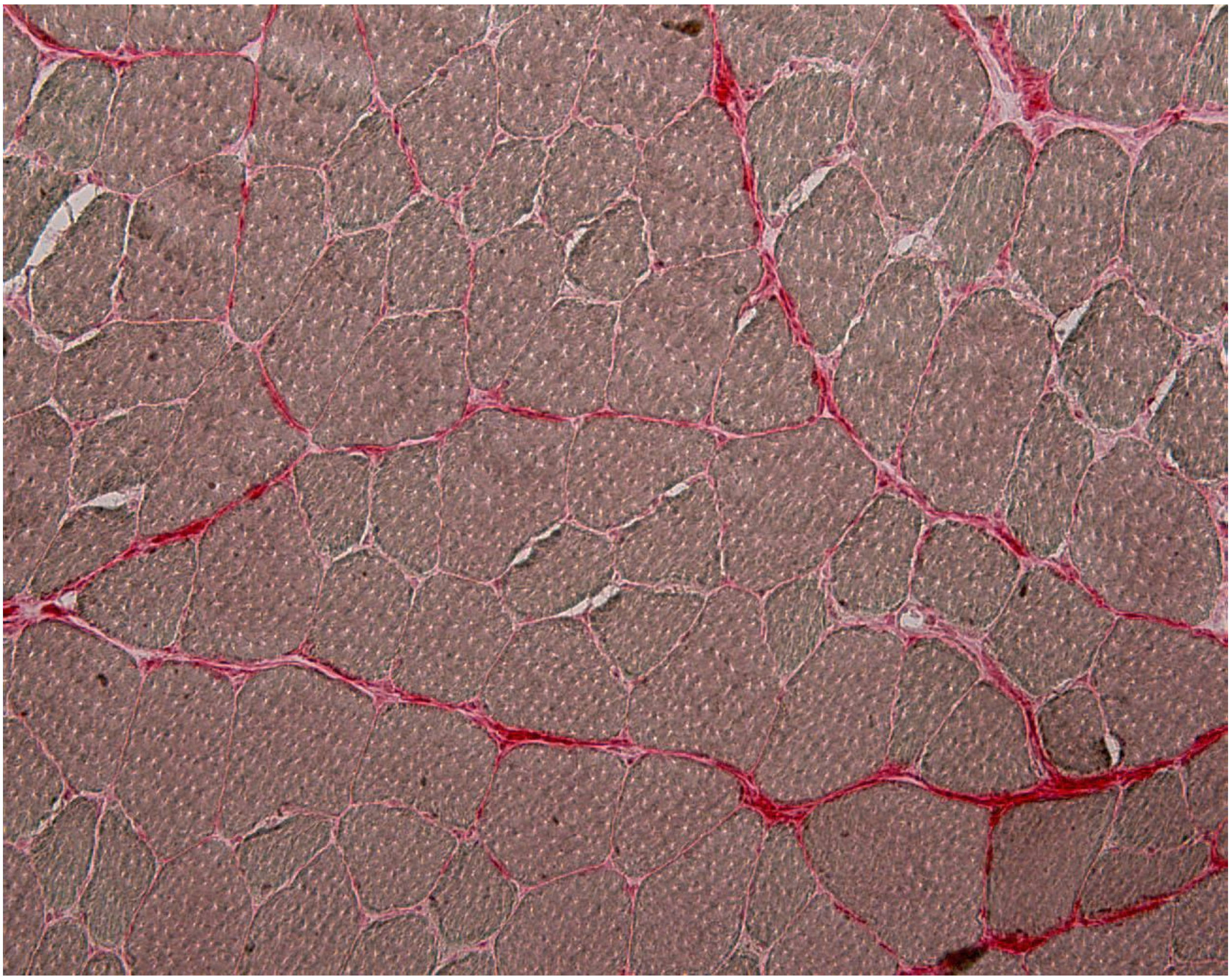

Figure S4. Representative image of collagen staining. EDL 7-day movement restriction

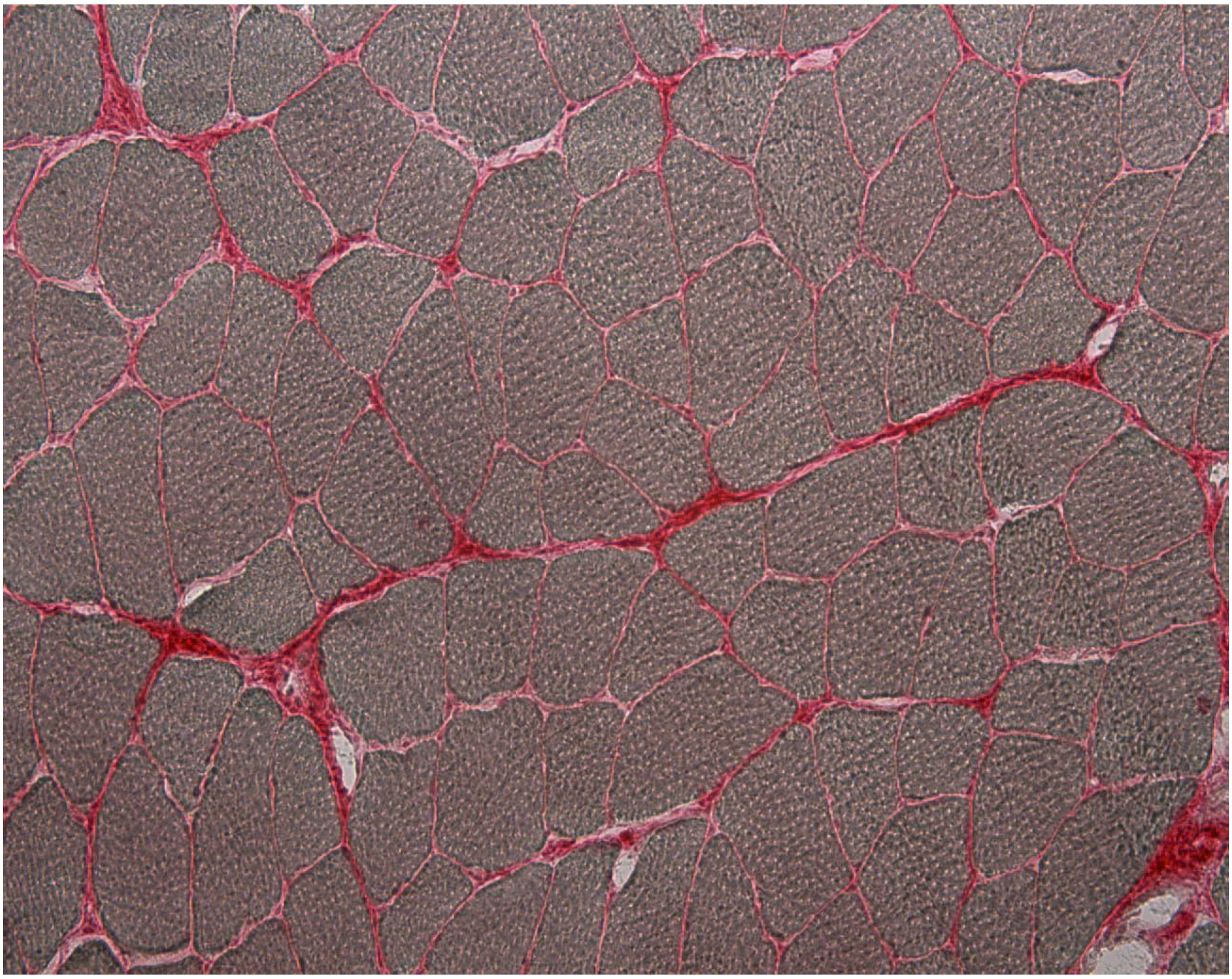

Figure S5. Representative image of collagen staining. Control 21-day movement restriction

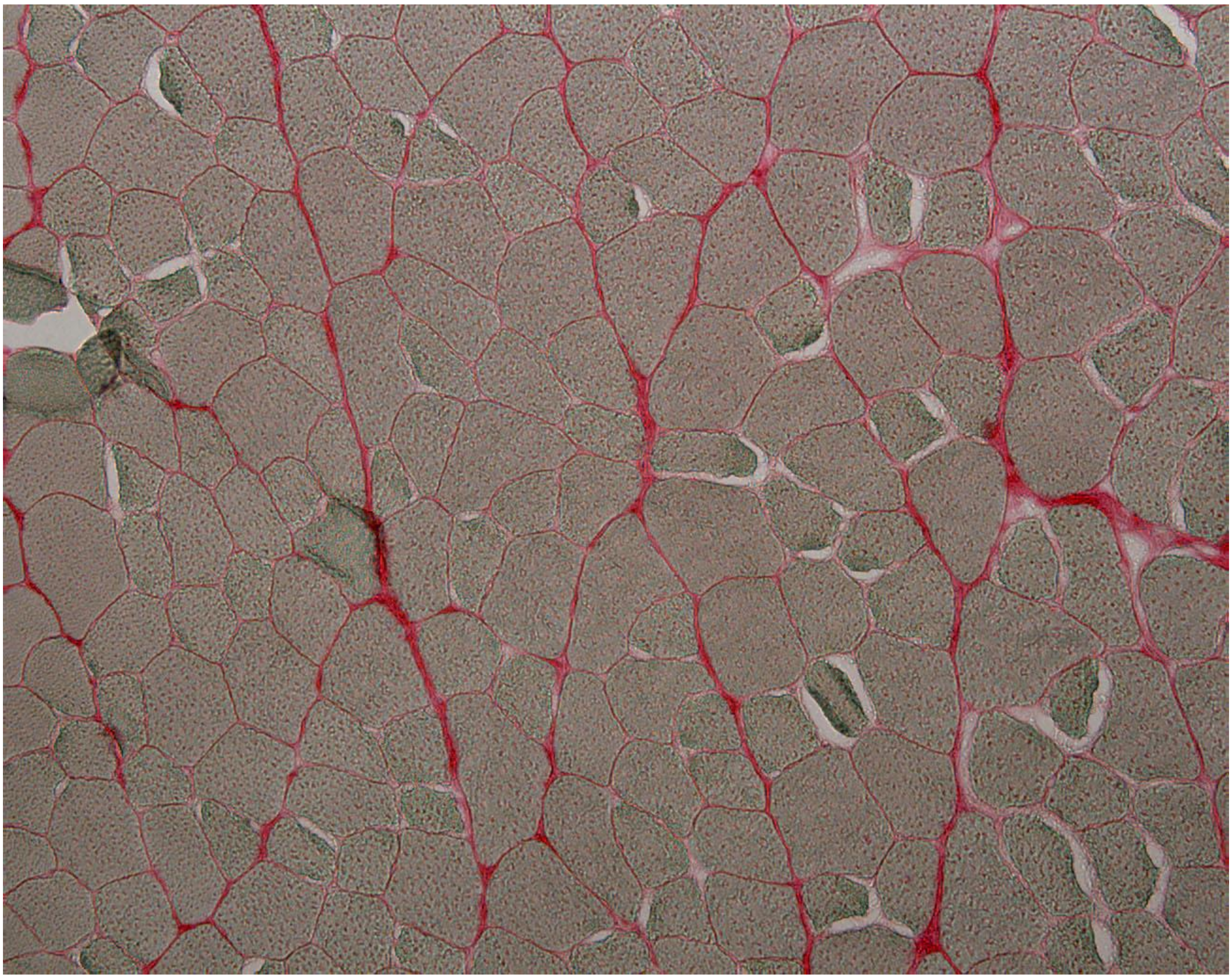

Figure S6. Representative image of collagen staining. EDL 21-day movement restriction
